# Potential of extracellular vesicles from human Wharton’s jelly and golden berries (*Physalis peruviana*) combined with polyvinyl alcohol/chitosan/fibroin hydrogel for wound healing: *In vitro* approaches on 1BR3 cell line

**DOI:** 10.5599/admet.3238

**Published:** 2026-04-10

**Authors:** Afrida Rizky Nurfajrin, Indra Wibowo, Anggraini Barlian

**Affiliations:** 1 Department of Biotechnology, Bandung Institute of Technology, Bandung, West Java, Indonesia; 2 Department of Biology, Bandung Institute of Technology, Bandung, West Java, Indonesia; 3 Scientific Imaging Center, Institut Teknologi Bandung, Bandung 40132, Indonesia

**Keywords:** Plant-derived exosome-like nanoparticles, mesenchymal stem cell, drug delivery, controlled release, fibroblast proliferation

## Abstract

**Background and purpose:**

The development of biocompatible delivery systems capable of enhancing wound healing remains a major challenge in drug delivery and regenerative medicine. Hydrogels represent promising wound dressings due to their ability to maintain a moist microenvironment, absorb exudates, and enable controlled release of bioactive agents. Recently, plant-derived exosome-like nanoparticles have emerged as a novel, non-toxic, and cross-kingdom therapeutic modality.

**Experimental approach:**

In this study, we investigated polyvinyl alcohol/chitosan/fibroin-based hydrogels as a delivery platform for plant-derived exosome-like nanoparticles isolated from *Physalis peruviana*, with human Wharton’s jelly mesenchymal stem cell-derived exosomes used as a biological comparator.

**Key results:**

plant-derived exosome-like nanoparticles isolated from *Physalis peruviana* and human Wharton’s jelly mesenchymal stem cell-derived exosomes were isolated and characterized in terms of size, morphology, and protein content. Composite hydrogels containing chitosan, polyvinyl alcohol, and fibroin (4 % and 10 %) were fabricated via freeze-thawing and characterized for hydrophilicity, swelling behaviour, water content, biodegradability, and protein release profiles. The biological performance of hydrogel-released media incorporating plant-derived exosome-like nanoparticles or human Wharton’s jelly mesenchymal stem cell-derived exosomes was evaluated *in vitro* using human dermal fibroblast 1BR3 cells through cytotoxicity, proliferation, and scratch migration assays. The fabricated hydrogels exhibited hydrophilic surfaces (contact angle < 90°), high swelling capacity (> 100 %), low water content, and gradual biodegradation over 10 days. Protein release peaked on day 3, indicating favourable release kinetics for bioactive delivery. All hydrogel formulations demonstrated good biocompatibility, with cell viability exceeding 75 %. Notably, hydrogels loaded with plant-derived exosome-like nanoparticles isolated from *Physalis peruviana* significantly promoted fibroblast proliferation and migration, with performance comparable to or exceeding that of human Wharton’s jelly mesenchymal stem cell-derived exosomes-loaded hydrogels, particularly in fibroin-containing formulations.

**Conclusion:**

These findings highlight the potential of *Physalis peruviana*-derived plant-derived exosome-like nanoparticles as a novel bioactive agent for wound healing and demonstrate that polyvinyl alcohol/chitosan/fibroin hydrogels serve as an effective delivery platform to enhance their biological activity. This study supports the translational potential of plant-derived exosome-like nanoparticles-based hydrogel systems for future wound healing applications.

## Introduction

The skin functions as the primary protective barrier of the human body, shielding internal tissues from mechanical injury, microbial invasion, and environmental stressors. Based on the duration and severity of tissue damage, wounds can be classified as acute or chronic. Currently, wounds represent a significant global health problem and are among the leading causes of morbidity and mortality worldwide, thereby exerting a substantial burden on public health systems and economic development [1,2]. Effective wound healing requires appropriate wound dressings that not only protect the wound area but also maintain a physiological microenvironment conducive to tissue regeneration [3].

Hydrogels have attracted considerable attention as wound dressings due to their hydrophilic nature, biocompatibility, biodegradability, and ability to maintain a moist environment that promotes cell proliferation and accelerates wound healing [1,4]. Structurally, hydrogels are three-dimensional polymeric networks that retain large amounts of water while maintaining mechanical integrity. These properties enable hydrogels to mimic the extracellular matrix (ECM), facilitate oxygen permeability, and facilitate functional modification, thereby supporting cellular activities essential for tissue repair [5]. Consequently, hydrogels are widely regarded as promising candidates for wound dressings and drug delivery systems.

Silk fibroin has been extensively studied as a biomaterial due to its outstanding mechanical strength, low immunogenicity, non-toxicity, biocompatibility, and biodegradability [6]. In addition, silk fibroin exhibits tuneable physicochemical properties, including adjustable elasticity, injectability, and environmental responsiveness, which enhance its applicability in tissue engineering and wound healing applications [7,8]. Composite hydrogels based on silk fibroin have demonstrated improved mechanical stability and biological performance, making them attractive materials for regenerative medicine.

Chitosan is another widely used biomaterial in biomedical applications due to its intrinsic bioactivity, biocompatibility, and biodegradability [9]. When incorporated into hydrogel systems, chitosan can enhance cellular responses and contribute antibacterial properties. Polyvinyl alcohol (PVA), on the other hand, is frequently added to hydrogel formulations due to its excellent water solubility, film-forming ability, and polymerization characteristics, which enhance the mechanical stability and structural integrity of hydrogels [10]. The combination of silk fibroin, chitosan, and PVA is therefore expected to yield composite hydrogels with favourable physicochemical and biological properties, suitable for wound-dressing applications.

In recent years, extracellular vesicles (EVs), particularly exosomes, have emerged as promising acellular therapeutic agents due to their role in mediating intercellular communication. Exosomes are lipid bilayer-enclosed vesicles that contain various bioactive molecules, including proteins, lipids, DNA, RNA, and microRNAs, and can modulate the behaviour of recipient cells [11]. MicroRNAs are short non-coding RNAs (17 to 24 nucleotides) that regulate post-transcriptional gene expression and play critical roles in cell proliferation, differentiation, and migration [11]. Mesenchymal stem cell-derived exosomes, including those obtained from human Wharton’s jelly, have been widely reported to promote wound healing by enhancing fibroblast proliferation, migration, and tissue remodelling.

In parallel with mammalian EV research, plant-derived exosome-like nanoparticles (PDENs) have attracted increasing interest as a novel class of bioactive nanocarriers. PDENs exhibit several advantageous characteristics, including non-toxicity, non-immunogenicity, biocompatibility, and the ability to mediate cross-kingdom biological communication [12]. These properties make PDENs particularly appealing for biomedical applications, including drug delivery and tissue regeneration.

*Physalis peruviana*, commonly known as ciplukan or golden berry, is a medicinal plant belonging to the *Solanaceae* family and is widely distributed in tropical and subtropical regions, including Indonesia [10]. The fruit of *Physalis peruviana* contains a variety of bioactive compounds, such as alkaloids, flavonoids (including rutin, kaempferol, and quercetin), carotenoids, phenolic compounds, vitamins (A, C, B1, B3, E and K), with anolides, and polysaccharides [7]. Among these compounds, carotenoids such as cryptoxanthin function as antioxidants, facilitate intercellular communication, and regulate immune responses [8]. Traditionally, *Physalis peruviana* has been used to treat various diseases, including diabetes, asthma, infections, kidney disorders, malaria, and wounds, owing to its antioxidant and anti-inflammatory properties [13,14]. Notably, the anti-inflammatory activity of *Physalis peruviana* has been reported to be comparable to that of hydrocortisone without inducing adverse effects such as skin atrophy, while simultaneously enhancing skin repair and remodelling processes [15].

Despite growing evidence supporting the therapeutic potential of PDENs, their application in wound healing, particularly when integrated into hydrogel-based delivery systems, remains limited. Moreover, the ability of hydrogels to serve as delivery platforms for PDENs and modulate their biological activity has not been comprehensively explored. Therefore, the present study aimed to develop PVA/chitosan/fibroin composite hydrogels as a delivery platform for PDENs isolated from *Physalis peruviana* (PENC) and to evaluate their *in vitro* wound healing potential. Exosomes derived from human Wharton’s jelly mesenchymal stem cells (hWJ-MSC-Exo) were included as a biological comparator. The hydrogels were characterized for physicochemical properties, biodegradability, and protein release behaviour, followed by *in vitro* assessment of cytotoxicity, fibroblast proliferation, and migration using the human dermal fibroblast 1BR3 cell line.

## Experimental

### Materials

This study was approved by the Ethics Committee of the Faculty of Medicine, Universitas Padjadjaran, Bandung, Indonesia (Ethical Clearance No. 535/UN6.KEP/EC/2024). Exosomes derived from human Wharton’s jelly mesenchymal stem cells were obtained from primary placental tissue cultures. The human dermal fibroblast cell line 1BR3 was used for all *in vitro* biological assays. Both hWJ-MSCs and 1BR3 cells were cultured in Dulbecco’s modified Eagle’s medium (DMEM, high glucose; Sigma-Aldrich, Germany) supplemented with 10 % foetal bovine serum (FBS; Gibco, Brazil) and 1 % antibiotic-antimycotic solution (penicillin 100 U mL^-1^ and streptomycin 100 μg mL^-1^; Gibco). Cells were maintained at 37 °C in a humidified incubator with 5 % CO_2_ and the culture medium was replaced every 2 to 3 days.

### Silk fibroin extraction

Silk fibroin was extracted from silkworm cocoons through a degumming process. Briefly, cocoons were boiled in a sodium bicarbonate (NaHCO_3_) solution for 1 h to remove the sericin layer, followed by thorough washing and drying for 24 h. The degummed fibres were then dissolved in calcium chloride dihydrate (CaCl_2_·2H_2_O) for 6 h. The resulting solution was dialyzed against deionized water for 3 days, with water replacement every 6 h, to remove residual salts. After dialysis, the fibroin solution was freeze-dried and subsequently redissolved in formic acid to obtain the desired concentrations.

### Fabrication of polyvinyl alcohol/chitosan/fibroin hydrogels

Chitosan solution (3 % w/v) was prepared by dissolving chitosan powder in formic acid under constant stirring. Polyvinyl alcohol (PVA) solution (10 % w/v) was prepared by dissolving PVA powder in distilled water at temperatures above 90 °C with continuous stirring. Glycerol solution (10 vol.%) was prepared using deionized water. Silk fibroin solutions were prepared at concentrations of 4 and 10 % (w/v).

Hydrogel formulations were prepared using fixed volumes of chitosan (3 % w/v), PVA (10 % w/v), and glycerol (10 vol.%), while silk fibroin concentration was varied at 4 % and 10 % (w/v). The mixtures were stirred for 30 min to ensure homogeneity and poured into Petri dishes. The hydrogel solutions were air-dried overnight in a fume hood, sealed, and subjected to a freeze–thawing process consisting of freezing at -80 °C for 20 h followed by thawing at 20 °C for 4 h. This cycle was repeated four times. Subsequently, the hydrogels were dried in a fume hood for 7 days to remove residual acid.

### Incorporation of extracellular vesicles into hydrogels

Extracellular vesicles (PENC or hWJ-MSC-Exo) were incorporated into the hydrogel system through a post-fabrication loading approach. Sterile hydrogel discs (1×1 cm^2^) were incubated in EV suspensions at predetermined concentrations for 24 h at 4 °C to allow passive absorption into the porous polymeric network. Following incubation, EV-loaded hydrogels were gently rinsed with sterile PBS to remove unbound vesicles. EVs were not embedded during hydrogel fabrication. Instead, hydrogels were incubated in EV-containing culture medium to obtain EV-enriched hydrogel-conditioned medium, which was subsequently used for biological evaluation.

### Contact angle measurement

The surface hydrophilicity of the hydrogels was evaluated by measuring the static water contact angle using the sessile drop method. A 4 μL droplet of distilled water was placed on the hydrogel surface, and the contact angle was recorded [16].

### Swelling ratio

Hydrogel samples were cut into 1×1 cm^2^ pieces and weighed to determine the initial dry weight (*W*_0_). The samples were immersed in 10 mL phosphate-buffered saline (PBS, pH 7.4) and incubated for 24 h. Excess surface liquid was gently removed, and the swollen weight (*W*_s_) was recorded. The equilibrium swelling ratio (ESR, %) was calculated using Equation (1) [17]:



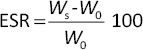

(1)


### Water content

Hydrogel samples were cut into 0.5×0.5 cm^2^ pieces and weighed to obtain the initial weight (*W*_s_). Samples were then dried in an oven for 24 h and reweighed (*W*_d_). Water content, % was calculated using Equation (2) [17]:



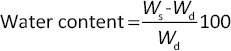

(2)


### Biogradation study

Hydrogel samples (1×1 cm^2^) were weighed to obtain the initial dry weight (W_0_) and immersed in PBS for 1, 3 and 7 days. At each time point, samples were removed, gently dried to remove excess surface moisture and weighed to obtain the remaining weight at time (W_*t*_). The degradation rate, % was calculated using Equation (3) [18]:



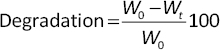

(3)


### Isolation of human Wharton’s jelly mesenchymal stem cells derived exosomes

hWJ-MSCs (passage 5) were cultured until approximately 60 % confluence. Cells were washed with PBS, and the culture medium was replaced with serum-free DMEM. After incubation for 48 h, the conditioned medium was collected and centrifuged at 2,000*g* for 30 min to remove cellular debris. Exosomes were isolated using the Invitrogen™ Total Exosome Isolation Kit according to the manufacturer’s instructions. Briefly, isolation reagent was added to the conditioned medium, incubated overnight at 4 °C, and centrifuged at 10,000*g* for 1 h. The resulting exosome pellet was resuspended in PBS [19]. PDEV morphology was characterized using a transmission electron microscope (TEM) (HT7700, 120 kV. Hitachi, Japan). Using the negative staining method. Dynamic light scattering (DLS) analysis was performed using a particle size analyser (Horiba, Japan SZ-100i) to measure the zeta potential of PDEV. Total PDEV protein concentration was measured using the Pierce™ BCA Protein Assay Kit (Thermo Fisher Scientific, USA) according to the manufacturer's protocol.

### Isolation of Physalis peruviana plant-derived exosome-like nanoparticles

Fresh Physalis peruviana fruits were obtained from a local market in Bandung, Indonesia. PDENs were isolated according to previously reported methods with slight modifications [19]. The fruits were washed, homogenized, and filtered sequentially through 100 μm and 40 μm nylon filters. The filtrate was centrifuged at 2,000*g* for 10 min, 6,000*g* for 20 min, and 10,000*g* for 40 min at 4 °C. Polyethylene glycol 6000 (PEG 6000) was added to the supernatant to a final concentration of 5 % (w/v), followed by overnight incubation at 4 °C. The mixture was centrifuged at 8,000*g* for 60 min to precipitate PDENs. The resulting pellet was resuspended in deionized water and filtered through a 0.22 μm polyethersulfone membrane. The PENC suspension was stored at -20 °C.

### Characterization of extracellular vesicles

The morphology of extracellular vesicles was examined using transmission electron microscopy (TEM; HT7700, Hitachi, Japan) with negative staining. Size distribution and concentration of hWJ-MSC-derived exosomes were analysed using nanoparticle tracking analysis (NTA; ViewSizer 3000, Horiba, Japan). The size distribution of PENC was evaluated using dynamic light scattering (DLS; SZ-100i, Horiba, Japan). Total protein concentration of vesicle samples was measured using a Pierce™ BCA Protein Assay Kit (Thermo Fisher Scientific, USA) according to the manufacturer’s protocol.

### Protein release assay

Hydrogel samples (1×1 cm^2^) were placed in a 12-well plate containing 3 mL PBS and incubated at 37 °C. At predetermined time points (days 1, 3 and 5), the supernatant was collected, and the protein concentration was determined using the Pierce™ BCA Protein Assay Kit. Absorbance was measured at 595 nm using a microplate reader.

### Biocompatible and cell proliferation assay

Hydrogel samples were sterilized by overnight UV exposure, followed by sequential immersion in 70 % ethanol and sterile PBS. The hydrogels were incubated in PBS overnight at 37 °C to remove residual ethanol and then incubated in culture medium to obtain hydrogel-released media corresponding to the formulations listed in Table 2. For cytotoxicity and proliferation assays, 1BR3 cells were seeded at 10,000 cells *per* well in 96-well plates and incubated for 24 h. The medium was replaced with a hydrogel-released medium.

**Table 1.** Treatments composition

Category	Content, %		
	Hydrogel fibroin	PENC	hWJ exosome
F4C2.5	4	2.5	-
F4C5	4	5	-
F10C2.5	10	2.5	-
F10C5	10	5	-
F4eH	4	-	2.5
F10eH	10	-	2.5

For cytotoxicity assessment, cells were incubated for 72 h. For proliferation analysis, cells were incubated for 1, 3, 5 and 7 days. After incubation, MTT reagent was added and incubated for 4 h. Formazan crystals were dissolved in dimethyl sulfoxide (DMSO), and absorbance was measured at 565 nm.

### Scratch migration assay

For migration analysis, 1BR3 cells were seeded at 20,000 cells *per* well in 24-well plates and cultured to confluence. The culture medium was replaced with hydrogel-released medium, and a linear scratch was created using a sterile 100 μL pipette tip. Cell migration was observed at 0, 12, 24, 36 and 48 h, and wound closure was quantified using ImageJ software [20].

### Statistical analysis

The results are reported as mean ± standard deviation (SD), *n* ≥ 3. The data were analysed using one-way ANOVA, with a *p*-value < 0.05 considered statistically significant. Statistical analysis was performed using GraphPad Prism software (GraphPad Software, Inc.).

## Results and discussion

### Characterization and multipotency of human Wharton’s jelly mesenchymal stem cells

**Figure 1.**
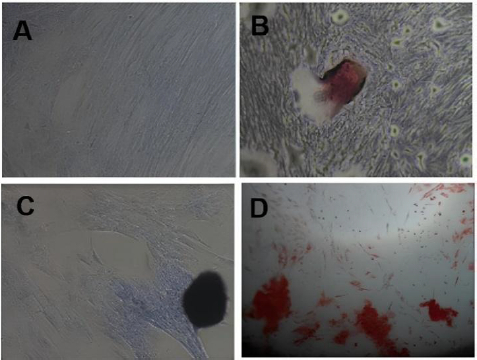
Multipotency assay of hWJ-MSC cultures induced with lineage-specific differentiation media. (A) Morphology of hWJ-MSCs without differentiation medium (standard culture medium), (B) Oil red O staining for adipocytes, (C) Alcian blue staining for chondrocytes and (D) Alizarin red staining for osteocytes

Primary cells isolated from human Wharton’s jelly successfully adhered to plastic culture surfaces and exhibited a fibroblast-like spindle morphology, characteristic of mesenchymal stem cells (MSCs) (Figure 1A). Trilineage differentiation assays demonstrated the multipotent capacity of the isolated cells. Adipogenic differentiation was confirmed by intracellular lipid droplet formation stained with Oil red O (Figure 1B), chondrogenic differentiation was indicated by Alcian blue positive proteoglycan deposition (Figure 1C), and osteogenic differentiation was evidenced by calcium mineralization stained with Alizarin red (Figure 1D).

**Figure 2.**
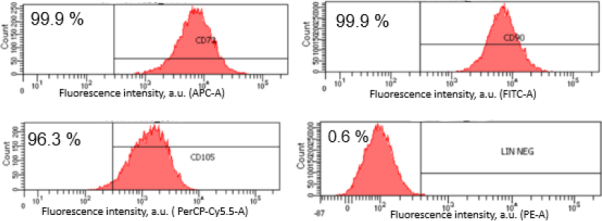
Flow cytometric analysis of surface marker expression in human Wharton’s jelly-derived mesenchymal stem cells (hWJ-MSCs). Histograms show the expression of positive mesenchymal markers CD73, CD90, and CD105, and the negative lineage marker (Lin^-^: CD34, CD45, CD11b, CD19 and HLA-DR). Fluorescence intensity was measured using APC-A (allophycocyanin), FITC-A (fluorescein isothiocyanate), PerCP-Cy5.5-A (peridinin chlorophyll protein-Cy5.5), and PE-A (phycoerythrin) channels. The x-axis represents fluorescence intensity (arbitrary units), and the y-axis represents cell count. Values are expressed as percentages of positive cells

Flow cytometric analysis further confirmed the MSC phenotype. The cells expressed high levels of positive mesenchymal markers CD90 (99.9 %), CD73 (99.9 %) and CD105 (96.3 %), while the negative lineage marker was minimally expressed (0.6 %) (Figure 2) These findings demonstrate that the isolated cells fulfilled the minimum criteria for MSCs as defined by the International Society for Cellular Therapy (ISCT) [21].

Flow cytometric analysis further confirmed the MSC phenotype. The cells expressed high levels of positive mesenchymal markers CD90 (99.9 %), CD73 (99.9 %) and CD105 (96.3 %), while the negative lineage marker was minimally expressed (0.6 %) (Figure 2) These findings demonstrate that the isolated cells fulfilled the minimum criteria for MSCs as defined by the International Society for Cellular Therapy (ISCT) [21].

### Fabrication of polyvinyl alcohol /chitosan/fibroin hydrogels

**Figure 3.**
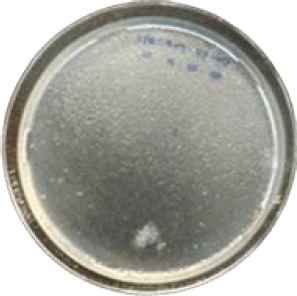
The result of PVA/Chitosan/Fibroin hydrogel fabrication using the four-cycle freeze-thaw method

Three hydrogel formulations were successfully fabricated using chitosan, PVA, glycerol, and varying concentrations of silk fibroin. The compositions included a control hydrogel (chitosan:PVA), fibroin 4 % (Fib4%), and fibroin 10 % (Fib10%). All hydrogels appeared transparent, yellowish-white, flexible, and mechanically stable, indicating successful polymer blending and crosslinking through the freeze-thaw method (Figure 3).

### Physical characterization of hydrogels

**Figure 4.**
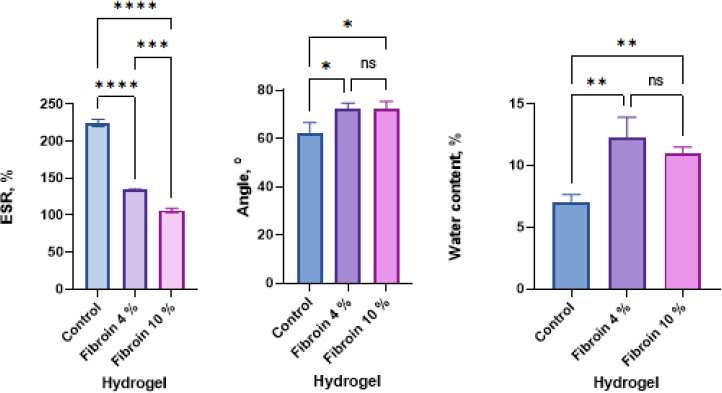
Graph of the physical characterization of PVA/chitosan/fibroin hydrogel: (a) swelling test graph, (b) contact angle measurement graph and (c) water content graph (*p < 0.05, **p < 0.01, ***p < 0.001, ****p < 0.0001, ns = not significant, *n* = 5)

**Figure 5.**
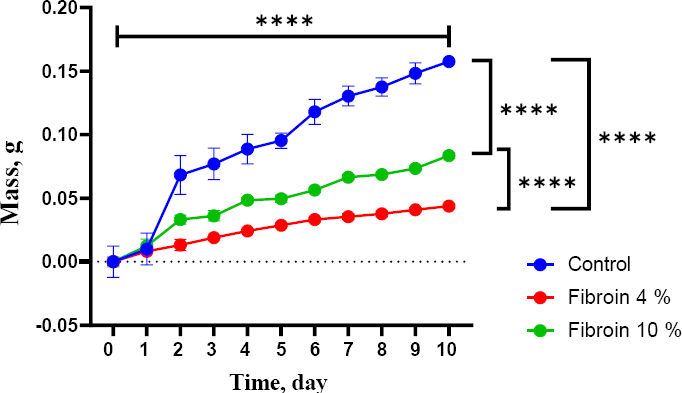
Graph of hydrogel mass reduction in PBS over a 10-day period, measured daily. Data are presented as mean ± standard deviation (****p < 0.0001, *n* = 5**)**

The fabricated hydrogels exhibited favourable physicochemical properties for wound dressing applications. Swelling ratios ranged from 106.8 to 224.7 %, indicating a high capacity for fluid absorption (Figure 4). Contact angle measurements ranged between 62.3 and 72.3°, confirming the hydrophilic nature of all hydrogel formulations. Water content values were relatively low, ranging from 6.9 to 12.3 %, suggesting adequate structural integrity while maintaining moisture retention. Biodegradation analysis revealed a gradual decrease in hydrogel mass over 10 days (Figure 5). All formulations showed progressive degradation, indicating their potential for natural resorption in biological environments.

### Isolation and characterization of extracellular vesicles

**Figure 6.**
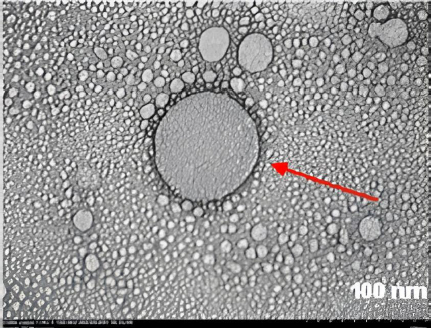
The morphology of PENC was observed using TEM. The red arrow indicates that PENC has a spherical morphology, and the scale bar represents 100 nm

Plant-derived exosome-like nanoparticles from *Physalis peruviana* (PENC) were successfully isolated using sequential centrifugation followed by PEG-6000 precipitation. Dynamic light scattering analysis revealed a particle size range of 171.9 to 174.6 nm with a polydispersity index of 0.112, indicating a homogeneous nanoparticle population. Transmission electron microscopy confirmed spherical morphology and membrane integrity (Figure 6). Protein concentrations of PENC ranged from 80 to 190 μg mL^-1^ as determined by BCA assay.

**Figure 7.**
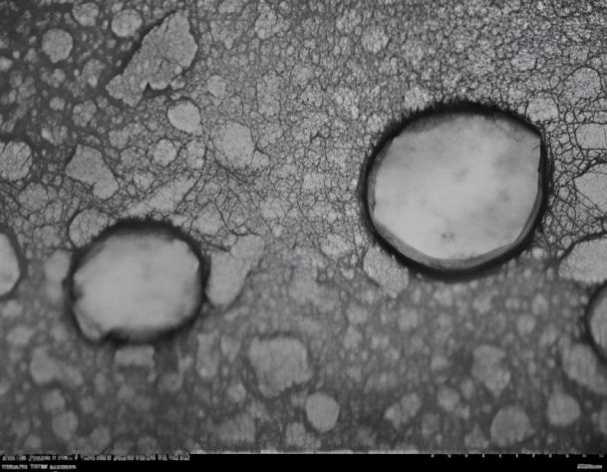
Characterization of hWJ-MSC exosomes using TEM. The red arrow indicates that the hWJ-MSC exosomes have a spherical morphology, and the scale bar represents 100 nm

**Figure 8.**
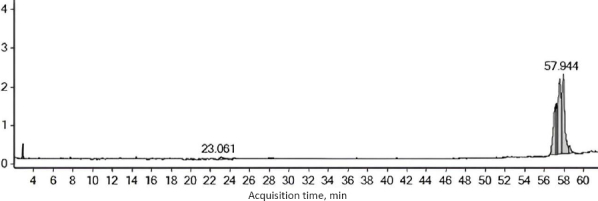
The results of PENC analysis using GC-MS. Significant compound peaks were observed at retention times of 23.061 minutes and 57.944 minutes. The peak at 57.944 minutes showed high intensity and indicated a dominant compound

Exosomes derived from hWJ-MSCs exhibited a mean size of approximately 94 nm with a modal size of 113 nm based on nanoparticle tracking analysis. TEM imaging revealed a typical spherical vesicular morphology (Figure 7). Protein concentrations ranged from 47 to 55 μg mL^-1^. GC-MS analysis of PENC identified seven major compounds, with dodecanoic acid, 1,2,3-propanetriyl ester as the most abundant component (Figure 8).

### Protein release profile of hydrogels

**Figure 9.**
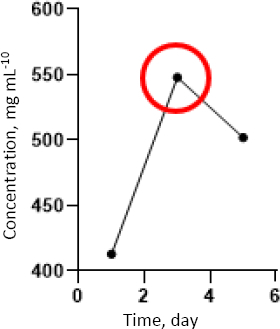
Graph of concentration measurements from the medium release assay of PVA/chitosan/fibroin hydrogel soaked in PBS for 1, 3 and 5 days. The red circle indicates the optimal medium release value during the test

Medium release assays showed a time-dependent pattern of protein release from the hydrogels (Figure 9). The highest protein concentration (549 μg mL^-1^) was observed on day 3, followed by a decline on day 5. These results indicate that day 3 represents the optimal release time point for biologically active components from the hydrogel matrix.

### Cytotoxicity and proliferation assay

**Figure 10.**
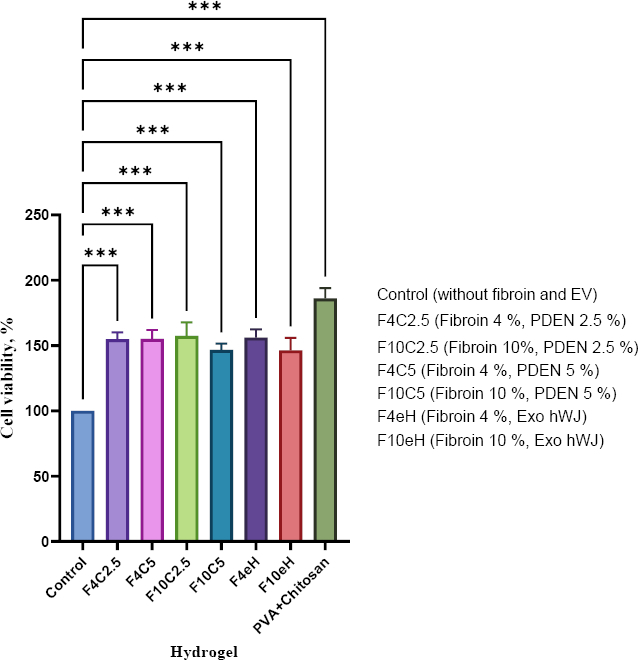
The cytotoxicity of EV combined with hydrogel on 1BR3 cell line was evaluated using the MTT assay. Cells were exposed to seven hydrogel medium release compositions for 48 hours. Data are presented as mean ± standard deviation (***p < 0.001, *n* = 5)

**Figure 11.**
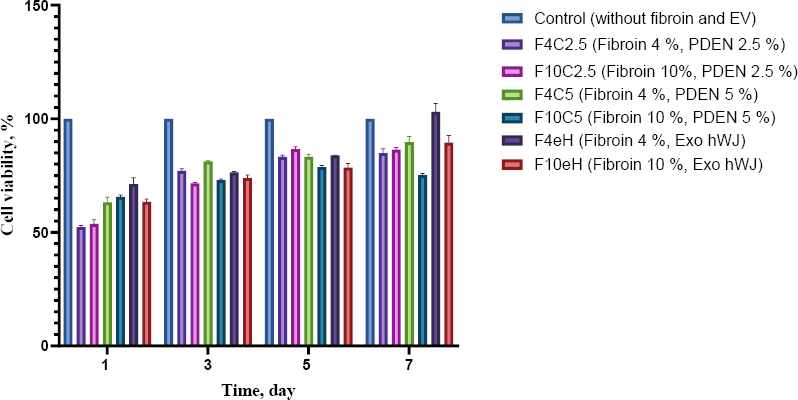
The effect of EV combined with hydrogel on 1BR3 cell proliferation was assessed using the MTT assay over 1, 3, 5 and 7 days. Cells were exposed to seven hydrogel medium release compositions for 7 days. Data are presented as mean ± standard deviation

MTT assays demonstrated that all hydrogel medium-release treatments maintained cell viability above 70 % after 48 h of exposure (Figure 10). According to ISO 10993-5, these results indicate non-cytotoxicity. Proliferation analysis over 1, 3, 5 and 7 days revealed that the F4eH, F4C5, and F10eH formulations significantly enhanced fibroblast proliferation compared to other groups (Figure 11).

### Cell migration analysis

**Figure 12.**
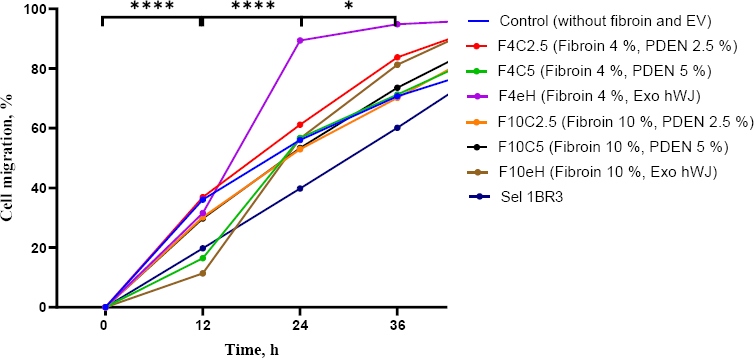
Migration assay of 1BR3 cell line induced by hydrogel composition with EV. Cells were exposed to seven hydrogel medium release combinations, scratched with a blue tip, and incubated for 48 hours (*p < 0.05, ****p < 0.0001, *n* =5 )

Scratch assays demonstrated enhanced fibroblast migration in all hydrogel-treated groups compared to the control (Figure 12). Notably, the F4eH, F4C2.5, and F10eH formulations exhibited the most pronounced wound closure at 36 hours, indicating accelerated migratory capacity.

### Discussion

The present study demonstrates that PVA/Chitosan/Fibroin-based hydrogels combined with extracellular vesicles, particularly PENC and hWJ-MSC-derived exosomes, exhibit physicochemical and biological properties favourable for wound-healing applications. The high swelling ratios observed across all hydrogel formulations are essential for effective wound exudate absorption and maintenance of a moist microenvironment, which is known to facilitate haemostasis and tissue regeneration [22,23]. The reduced swelling observed at higher fibroin concentrations may be attributed to the greater abundance of hydrophobic amino acid residues in fibroin, which restrict water penetration within the polymer network [24]. Contact angle values below 90° further confirm the hydrophilic nature of the hydrogels, supporting cell attachment and migration [23].

Controlled biodegradation over 10 days suggests that the hydrogels can provide temporary structural support while gradually resorbing, a critical requirement for wound dressings and drug delivery systems [25]. The degradation mechanism is likely driven by water diffusion into the hydrogel matrix, disrupting hydrogen bonding and relaxing polymer chains.

Successful isolation of PENC and hWJ-MSC-derived exosomes with appropriate size, morphology, and protein content confirms their suitability as bioactive agents. The size range of PENC aligns with previously reported PDENs, which are known to exhibit cross-kingdom bioactivity, low immunogenicity, and high biocompatibility [24,26]. The presence of lipid esters and fatty acid derivatives identified by GC-MS may contribute to PENC's biological activity, particularly by modulating inflammation and cell migration [27].

The protein release profile indicates that the hydrogel matrix functions as an effective delivery system, with peak release occurring on day 3. This sustained release behaviour is advantageous for wound healing, as it enables prolonged exposure of cells to bioactive molecules, including proteins derived from fibroin, chitosan, and incorporated EVs [28-30]. Biocompatibility assays confirmed that all hydrogel formulations were non-toxic, with cell viability exceeding the acceptable threshold of 70 % [31]. Enhanced proliferation observed in the F4eH, F4C5, and F10eH formulations may be attributed to the synergistic effects of fibroin-derived peptides, antioxidant compounds present in PENC, and regulatory cargo, such as miRNAs and proteins, carried by hWJ-MSC exosomes [32,33]. However, reduced proliferation at higher fibroin concentrations over extended culture periods may be associated with residual sericin content, which has been reported to inhibit cell proliferation under certain conditions [34].

The protein release profile obtained from EV-loaded hydrogels demonstrated a time-dependent pattern, with a peak concentration observed on day 3. Importantly, this release behaviour is not merely a physicochemical characteristic of the hydrogel matrix but represents a functional indicator of extracellular vesicle bioavailability. Extracellular vesicles are known to contain membrane-associated proteins, growth factors, lipids, and regulatory RNAs that mediate intercellular communication and tissue repair processes [35,36]. Therefore, the total protein quantified in the release medium may, at least in part, reflect the release kinetics of EV-associated bioactive cargo from the polymeric network.

Notably, the peak protein release observed on day 3 temporally corresponded with enhanced fibroblast proliferation and accelerated wound closure observed in EV-loaded hydrogel groups. Mesenchymal stem cell-derived exosomes have been widely reported to promote fibroblast proliferation, migration, and angiogenesis during cutaneous wound repair [37]. This temporal association suggests that sustained EV release directly modulates fibroblast behaviour.

The early release phase within the first 36 hours in the scratch assay indicates that bioactive molecules released from the hydrogel matrix actively participate in cytoskeletal reorganization and extracellular matrix remodelling. Previous studies have demonstrated that incorporating EVs into hydrogel systems enhances retention, stability, and sustained bioactivity, thereby improving wound-healing outcomes [4].

In the context of plant-derived vesicles, PDENs have been reported to exhibit cross-kingdom biological activity and anti-inflammatory properties, supporting tissue regeneration and cellular proliferation [12,31]. Therefore, the controlled protein release observed in this study likely represents sustained delivery of pro-regenerative signals that enhance cellular responses critical for wound healing.

Taken together, these findings support the concept that the protein release profile serves as a surrogate marker of EV-mediated therapeutic activity. The hydrogel matrix functions not only as a structural scaffold but also as a controlled delivery platform that modulates EV bioavailability, thereby directly influencing fibroblast proliferation and migration—two fundamental cellular processes underlying cutaneous wound repair

Cell migration assays further demonstrated the pro-regenerative potential of EV-loaded hydrogels. The enhanced migratory response, particularly in PENC-containing formulations, suggests that PDENs actively modulate signalling pathways involved in cytoskeletal reorganization and extracellular matrix remodelling [28,29]. These findings highlight the ability of plant-derived and stem cell-derived vesicles to accelerate wound closure through complementary mechanisms.

Overall, the integration of EVs into PVA/Chitosan/Fibroin hydrogels represents a promising strategy for advanced wound dressings with controlled release, high biocompatibility, and enhanced cellular responses relevant to skin regeneration.

## Conclusions

This study successfully developed PVA/chitosan/fibroin composite hydrogels as delivery platforms for plant-derived exosome-like nanoparticles isolated from *Physalis peruviana*. The hydrogels exhibited favourable physicochemical properties, including hydrophilicity, high swelling capacity, controlled biodegradation, and sustained protein release. *In vitro* biological evaluation demonstrated that PENC-loaded hydrogels were biocompatible and effectively enhanced fibroblast proliferation and migration, with performance comparable to or exceeding that of hWJ-MSC-derived exosome-loaded hydrogels. These findings highlight the potential of PDENs as a viable, scalable alternative to mammalian exosomes for wound-healing applications. The integration of PDENs into a hydrogel-based delivery system provides a promising strategy for localized, sustained delivery of bioactive nanoparticles, supporting their future development in regenerative medicine and drug delivery. The sustained protein-release profile was functionally associated with enhanced fibroblast proliferation and migration, underscoring the importance of controlled EV delivery in wound-healing applications.
